# Angiotensin receptor-neprilysin inhibitor delays progression from paroxysmal to persistent atrial fibrillation

**DOI:** 10.1038/s41598-023-30349-w

**Published:** 2023-02-23

**Authors:** Youzheng Dong, Zhenyu Zhai, Jihong Wang, Zhen Xia, Zirong Xia, Bo Zhu, Quanbing Dong, Qing Li, Juxiang Li

**Affiliations:** Department of Cardiovascular Medicine, The Second Affiliated Hospital of Nanchang University, No.1 of Minde Road, Nanchang, 330006 China

**Keywords:** Cardiology, Medical research

## Abstract

Progression from paroxysmal to persistent atrial fibrillation (AF) is linked to adverse clinical outcomes. The present study sought to clarify whether angiotensin receptor-neprilysin inhibitor (ARNI) can delay AF progression. A retrospective cohort study was conducted on consecutive patients with paroxysmal AF admitted at the Second Affiliated Hospital of Nanchang University between January 2017 and January 2022. The risk of AF progression from paroxysmal to persistent was compared between paroxysmal patients treated with ARNI and those who received an angiotensin receptor blocker (ARB). Seven-day Holter monitoring was performed to identify persistent AF. Propensity-score matched analysis was performed to compare the two groups. Cox-regression was used to estimate the hazard ratio (HR) for AF progression events. A total of 1083 patients were screened, and 113 patients in the ARB group and 57 patients in the ARNI group were eligible for analysis. Before propensity-score matching, the ARNI therapy was associated with a lower risk of AF progression than the ARB therapy (HR 0.34; 95% confidence interval [CI] 0.14–0.81; P = 0.015) after a median follow-up of 705 (interquartile range [IQR] 512 to 895) days. Among 170 patients, 47 ARNI-treated patients were successfully matched to 47 ARB-treated patients. After a median follow-up of 724 (541–929) days, compared to ARB, ARNI significantly reduced the risk of AF progression (HR 0.32; 95% CI 0.12–0.88; P = 0.016). ARNI may be superior to ARB in reducing the risk of progression from paroxysmal to persistent AF.

## Introduction

Currently, the prevailing classification of atrial fibrillation (AF) is based on the duration and spontaneous termination of AF episodes. Paroxysmal AF is defined as AF episodes that terminated spontaneously within 7 days of onset, while persistent AF describes AF episodes lasting longer than 7 days and less than 12 months^1^. Paroxysmal AF is considered the early stage of the natural history of AF. Most patients inevitably progress from brief, rare episodes of AF to long-term, frequent episodes, which are associated with risk factors, including age, left atrial size, hypertension, diabetes, and heart failure (HF), even with drug control^2^. This progression is frequently characterized by deteriorating atrial remodeling and is associated with adverse cardiovascular events, hospitalizations, and death^3^. Moreover, both medical therapy and radiofrequency ablation are significantly less effective in persistent AF than in paroxysmal AF. Therefore, delaying AF progression is a highly attractive management strategy to improve the prognosis of patients with paroxysmal AF.

Angiotensin receptor-neprilysin inhibitor (ARNI, sacubitril/valsartan), a novel single co-crystal, is composed of sacubitril and valsartan in a ratio of 1:1^4^. Sacubitril is a prodrug that is converted to an active metabolite, LBQ657, which can inhibit the activity of neutral endopeptidase, thereby elevating the levels of natriuretic peptides with antihypertensive and organ-protective effects in vivo^4^. Valsartan, a traditional angiotensin II type 1 receptor inhibitor, also has antihypertensive and anti-cardiac remodeling effects. The co-crystal structure of sacubitril/valsartan ensures synchronization in the absorption and elimination of sacubitril and valsartan, which generates a synergistic effect of cardiovascular benefits^5^. In the last decade, numerous well-designed clinical studies have been conducted to verify whether sacubitril/valsartan with dual effect is superior to conventional renin–angiotensin–aldosterone system (RAAS) inhibitors. Sacubitril/valsartan further reduced the risk of hospitalization for HF and death in HF patients with left ventricular ejection fraction (LVEF) ≤ 40%^6^. Compared with olmesartan, sacubitril/valsartan significantly reduced 24-h ambulate blood pressure in patients with hypertension^7,8^. In addition, sacubitril/valsartan appears to improve outcomes in patients with myocardial infarction (MI)^9–11^. However, few studies have reported the effects of sacubitril/valsartan on AF. Herein, we examined whether sacubitril/valsartan could inhibit the progression of paroxysmal AF.

## Results

### Characteristics of the study population

**Figure 1:**
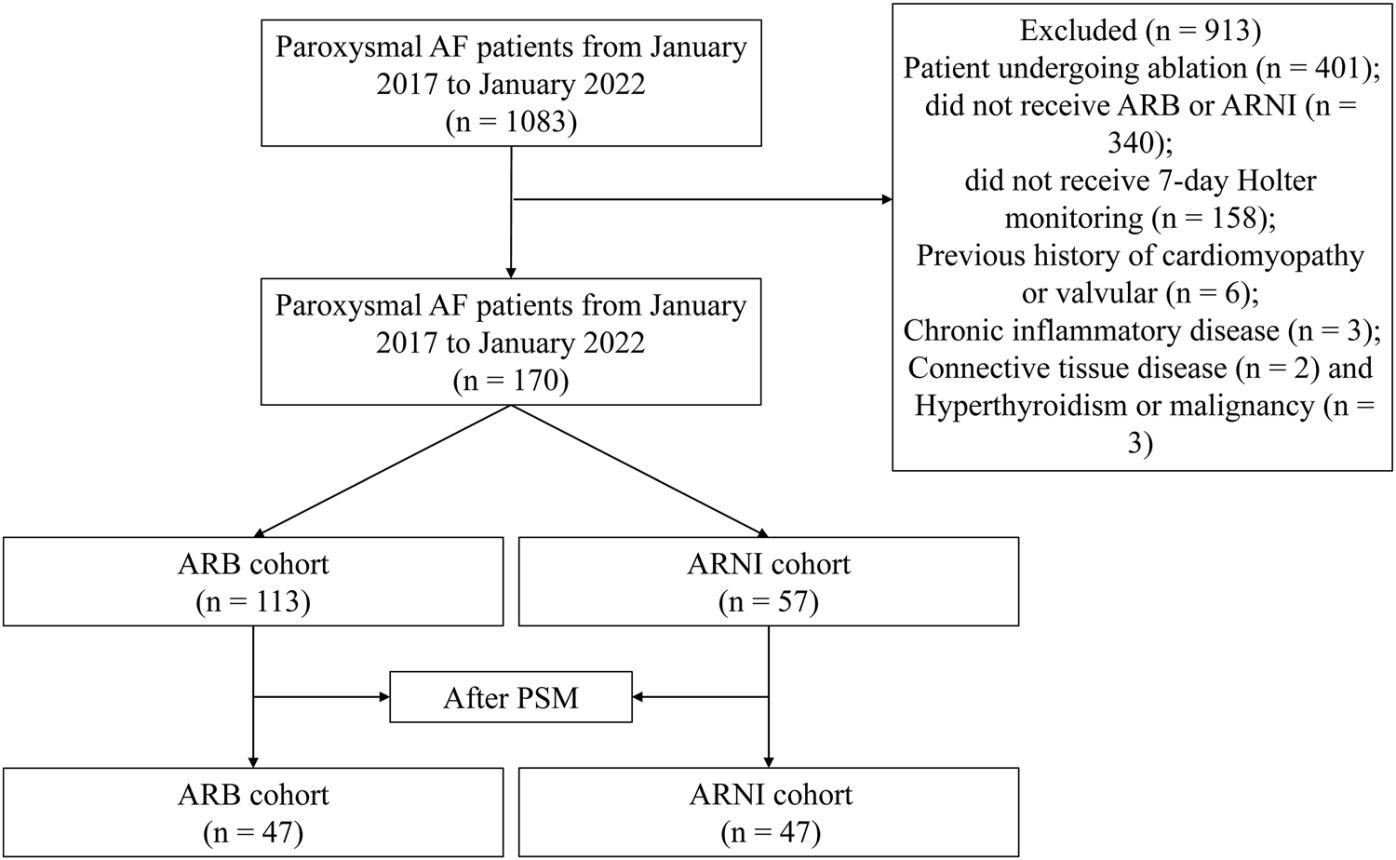
Study flow diagram. *AF* atrial fibrillation, *ARB* angiotensin receptor blocker, *ARNI* angiotensin receptor-neprilysin inhibitor, *PSM* propensity score matching.

A total of 1083 patients diagnosed with paroxysmal AF were identified, of which 170 patients were eligible for analysis (Fig. 1). Of the 170 patients, 113 (66.5%) patients received angiotensin receptor blocker (ARB) and 57 (33.5%) received ARNI. Before propensity-score matching (PSM), baseline characteristics such as age, total cholesterol (TC), low density lipoprotein cholesterol (LDL-c), brain natriuretic peptide (BNP), left atrial diameter (LAD), LVEF, and history of HF significantly differed between ARB and ARNI groups (Table 1). However, no notable difference in these baseline characteristics was detected between the two groups after PSM. Of the 47 ARB-treated patients, the average age was 64.2 years, 66.0% were males, and the average body mass index (BMI) was 24.9. In the ARNI group, the average age was 64.2 years, 68.1% were males, and the average BMI was 24.7. Moreover, 12 (27.3%) patients in the ARB group and 10 (22.7%) in the ARNI group reached the target dose during follow-up. Types of drugs used in the ARB cohort are shown in Table 2. The time interval for each Holter monitoring from the both groups are shown in Supplementary Tables S1 and S2.

**Table 1:** Baseline characteristics of the study population.

Characteristic	Before matching			After matching		
	ARB	ARNI	*P*-value	ARB	ARNI	*P*-value
N	113	57		47	47	
Age (years)	60.82 (10.61)	64.35 (10.46)	0.041	64.19 (7.57)	64.17 (10.49)	0.991
Male (%)	78 (69.03%)	41 (71.93%)	0.697	31 (65.96%)	32 (68.09%)	0.826
BMI (kg/m^2^)	25.34 (3.01)	24.40 (3.18)	0.061	24.85 (3.26)	24.66 (3.19)	0.779
Duration of AF (months)	12.00 (6.00–48.00)	13.00 (3.00–48.00)	0.695	24.00 (6.00–36.00)	22.00 (4.00–48.00)	0.677
Hypertension (%)	85 (75.22%)	40 (70.18%)	0.481	36 (76.60%)	33 (70.21%)	0.484
Hyperlipidemia (%)	38 (33.63%)	16 (28.07%)	0.462	16 (34.04%)	15 (31.91%)	0.826
Diabetes mellitus (%)	21 (18.58%)	13 (22.81%)	0.516	11 (23.40%)	11 (23.40%)	1.000
CAD (%)	17 (15.04%)	10 (17.54%)	0.674	8 (17.02%)	8 (17.02%)	1.000
HF (%)	22 (19.47%)	20 (35.09%)	0.026	12 (25.53%)	15 (31.91%)	0.494
Current smoking (%)	21 (18.58%)	9 (15.79%)	0.652	7 (14.89%)	6 (12.77%)	0.765
Current alcohol (%)	19 (16.81%)	11 (19.30%)	0.688	8 (17.02%)	8 (17.02%)	1.000
SBP (mm Hg)	132.09 (20.87)	130.70 (20.05)	0.679	135.74 (20.12)	131.45 (19.85)	0.300
DBP (mm Hg)	76.73 (12.45)	75.47 (10.41)	0.512	76.02 (11.69)	77.02 (9.19)	0.646
TC (mmol/L)	4.62 (1.04)	4.95 (1.00)	0.044	4.99 (1.05)	4.99 (1.01)	0.976
TG (mmol/L)	1.75 (1.57)	1.79 (2.00)	0.878	1.87 (1.65)	1.90 (2.17)	0.947
HDL-c (mmol/L)	1.08 (0.36)	1.00 (0.33)	0.161	1.01 (0.33)	1.01 (0.34)	0.973
LDL-c (mmol/L)	2.53 (0.76)	2.78 (0.69)	0.042	2.83 (0.78)	2.77 (0.69)	0.736
eGFR (mL/min per 1.73m^2^)	82.69 (19.19)	80.04 (22.66)	0.426	80.85 (19.08)	80.00 (20.34)	0.835
BNP (pg/ml)	94.30 (36.51–190.53)	221.04 (53.26–461.75)	0.012	110.56 (41.02–400.14)	155.68 (49.08–394.78)	0.951
LAD (mm)	36.63 (5.39)	38.91 (6.57)	0.017	37.23 (5.61)	37.79 (5.79)	0.639
LVEF (%)	62.52 (8.57)	57.79 (11.42)	0.003	61.21 (10.82)	59.91 (10.73)	0.561
NYHA functional class (%)			0.080			0.494
I	91 (80.53%)	37 (64.91%)		35 (74.47%)	32 (68.09%)	
II	22 (19.47%)	20 (35.09%)		12 (25.53%)	15 (31.91%)	
CHA_2_DS_2_-VASc score	2.25 (1.45)	2.40 (1.36)	0.501	2.38 (1.42)	2.43 (1.36)	0.883
Baseline medications (%)						
Beta-blockers	55 (48.67%)	29 (50.88%)	0.786	22 (46.81%)	22 (46.81%)	1.000
CCB	39 (34.51%)	20 (35.09%)	0.941	15 (31.91%)	14 (29.79%)	0.823
Statins	35 (30.97%)	19 (33.33%)	0.755	17 (36.17%)	15 (31.91%)	0.663
MRA	10 (8.85%)	8 (14.04%)	0.300	6 (12.77%)	6 (12.77%)	1.000
Diuretics	32 (28.32%)	20 (35.09%)	0.366	18 (38.30%)	18 (38.30%)	1.000
Anticoagulation			0.613			0.933
Warfarin	6 (5.31%)	2 (3.51%)		2 (4.26%)	1 (2.13%)	
Dabigatran	25 (22.12%)	17 (29.82%)		12 (25.53%)	13 (27.66%)	
Rivaroxaban	42 (37.17%)	22 (38.60%)		18 (38.30%)	19 (40.43%)	
AADs			0.200			1.000
Class I	25 (22.12%)	17 (29.82%)		14 (29.79%)	14 (29.79%)	
Class III	11 (9.73%)	9 (15.79%)		5 (10.64%)	5 (10.64%)	
Follow-up (days)	684.00 (487.00–884.00)	726.00 (544.00–910.00)	0.328	743.00 (529.00–935.50)	709.00 (542.50–885.50)	0.991

Data are presented as mean ± standard deviation, or median (interquartile range) and percentages. *ARB* angiotensin receptor blocker, *ARNI* Angiotensin receptor-neprilysin inhibitor, *BMI* body mass index, *AF* atrial fibrillation, *CAD* coronary artery disease, *HF* Heart failure, *SBP* systolic blood pressure, *DBP* diastolic blood pressure, *TC* total cholesterol, *TG* triglyceride, *HDL-c* high density lipoprotein cholesterol, *LDL-c* low density lipoprotein cholesterol, *eGFR* estimated glomerular filtration rate, *BNP* brain natriuretic peptide, *LAD* left atrial diameter, *LVEF* left ventricle ejection fraction, *NYHA* New York Heart Association, *CCB* calcium channel blockers, *MRA* mineralocorticoid receptor antagonist, *AADs* antiarrhythmic drugs.

**Table 2:** Type of ARB prescribed in the matched cohort.

	N
Valsartan	31 (66.0%)
Irbesartan	11 (23.4%)
Losartan	5 (10.6%)

*ARB* angiotensin receptor blocker.

### Primary endpoint

**Figure 2:**
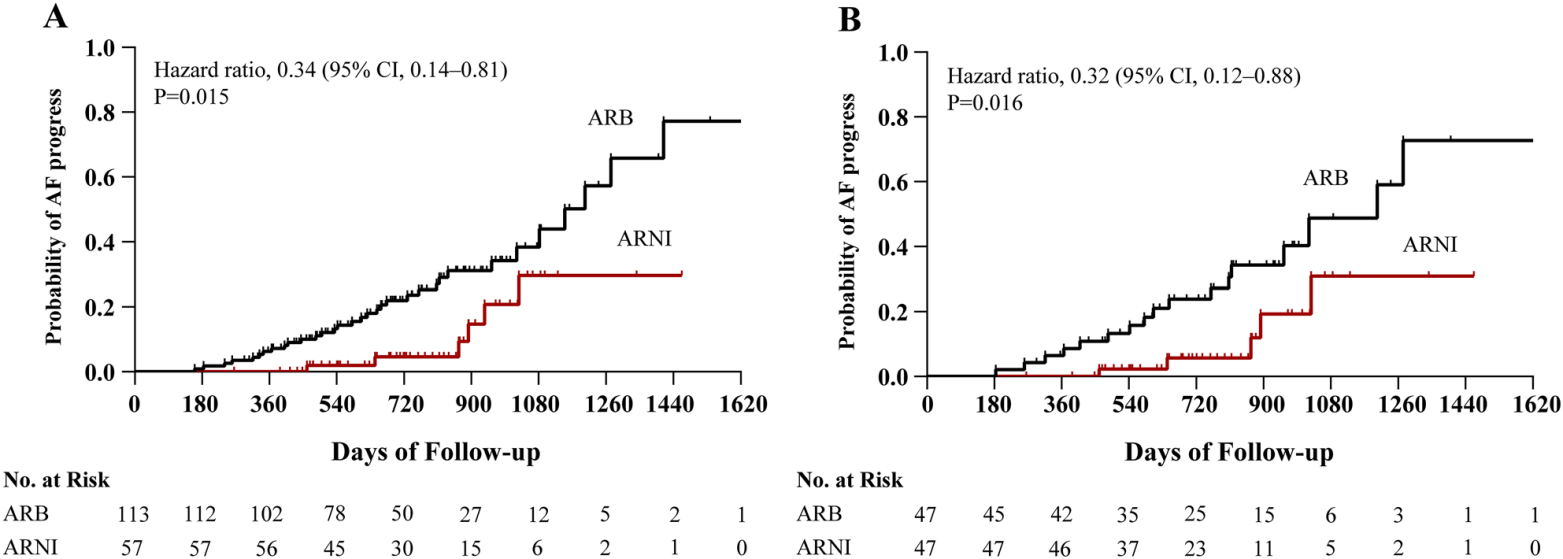
Kaplan–Meier curves for AF progression before PSM (**A**) and after PSM (**B**). *AF* atrial fibrillation, *ARB* angiotensin receptor blocker, *ARNI* angiotensin receptor-neprilysin inhibitor, *PSM* propensity score matching, *CI* confidence interval.

Before PSM, 33 (29.2%) patients in the ARB cohort and 6 (10.5%) patients in the ARNI had persistent AF after a median follow-up of 684 (interquartile range [IQR] 487–884) and 726 (IQR 544–910) days, respectively. Figure 2A shows the Kaplan–Meier curve of AF progression. It was found that compared with ARB, ARNI treatment significantly reduced the risk of AF progression (hazard ratio [HR] 0.34; 95% confidence interval [CI] 0.14–0.81; P = 0.015; Table 3). After PSM, the median follow-up time and the occurrence of persistent AF were 743 (IQR 529–936) days and 17 (36.2%) and 709 (IQR 543–886) days and 5 (10.6%) in ARB and ARNI groups, respectively. Relative to those patients with ARB, the HR for AF progression in those patients with ARNI was 0.32 (95% CI 0.12–0.88; P = 0.016; Fig. 2B and Table 3). The changes in LVEF and LAD of the two groups before and after treatment are shown in Supplementary Table S3.

**Table 3:** Risk of AF progression in the original cohort and the matched cohort.

	No. of patients with event	Event rate	HR (95% CI)	*P*
Before matching				
ARB	33	29.2%	Ref.	
ARNI	6	10.5%	0.34 (0.14, 0.81)	0.015
After matching				
ARB	17	36.2%	Ref.	
ARNI	5	10.6%	0.32 (0.12, 0.88)	0.016

*AF* atrial fibrillation, *ARB* angiotensin receptor blocker, *ARNI* Angiotensin receptor- neprilysin inhibitor, *HR* hazard ratio, *CI* confidence interval.

## Discussion

The present study explores for the first time the efficacy of ARNI in patients with paroxysmal AF. It was found that compared with ARB, ARNI treatment substantially reduced the risk of progression from paroxysmal to persistent AF. This finding may be valuable in guiding AF management.

Currently, antiarrhythmic drugs (AADs) and energy ablation are considered the primary rhythm control strategy for patients with paroxysmal AF. According to the 2020 European Society of Cardiology (ESC) AF guidelines, AADs are recommended for the ‘general’ paroxysmal AF population, while ablation is recommended for paroxysmal AF patients with HF with reduced ejection fraction^1^. However, both AADs and ablation therapies are faced with a high risk of AF recurrence and progression in the future due to advance atrial remodeling and persistent risk factors^12^. A previous study involving 1219 patients showed that progression of AF occurred in 178 (15%) patients at 12 months^13^. Another large cohort study demonstrated that the risk of progression was 8.6% and 24.7% at 12 months and 5 years, respectively^14^. Other studies reported that the transition from paroxysmal to persistent AF is highly variable, ranging from 2 to 34% in 1 year^12^.

Substantial evidence indicates that sacubitril/valsartan is a promising drug against HF and hypertension. Compared with traditional RAAS inhibitors, it exhibited a better anti-ventricular remodeling effect and antihypertensive effect due to its dual synergistic effect. Logically, patients with MI or AF are also likely to benefit from sacubitril/valsartan. However, the recent PARADISE-MI study found that sacubitril/valsartan did not achieve statistical significance in reducing cardiovascular death or overall HF in patients with acute MI^15^. Nevertheless, several clinical studies have shown that sacubitril/valsartan is superior to traditional RAAS inhibitors in improving atrial structural remodeling and reducing AF recurrence in patients with AF after catheter ablation^16,17^.

In the era of precision medicine, AF progression is a concern in AF management. AF-, age-, and other disease-related remodeling all promote AF progression. Cardiac electrical remodeling together with structural remodeling was implicated in this incremental process. The key electrical remodeling mainly involves alterations in calcium handling that cause triggered activity, changes in sodium channel function that lead to slowed conduction, and alterations in ionic current that facilitate reentry by shortening the action potential duration (APD)/refractory period^18^. Previous studies have indicated that pulmonary vein ectopic activity is associated with the occurrence of early afterdepolarizations (EADs) and delayed afterdepolarizations (DADs)^19^. EADs predominantly occur in the context of prolonged APD, and the prolongation of APD occurs as a result of both increased inward current of depolarization (persistent Na^+^ current, late Na^+^ current, and L-type Ca^2+^ current ([I_CaL_]) and decreased outward K^+^ current of repolarization^20,21^. DADs are generally derived from Ca^2+^-handling abnormalities, including anomalous sarcoplasmic reticulum (SR) Ca^2+^ release and spontaneous diastolic SR Ca^2+^ leakage, and are enhanced by SR Ca^2+^ overload and ryanodine receptor 2 (RyR2) dysfunction^22^. Increased cytosolic Ca^2+^ levels during diastole can result in sodium-calcium exchanger (NCX) hyperfunction that leads to a transient-inward current, which can cause membrane depolarization, triggering arrhythmias^22^. A recent study showed that sacubitril/valsartan could ameliorate the dysfunction of the RyR2 complex and NCX1 complex, which suggesting that sacubitril/valsartan may improve SR Ca^2+^ mishandling and help reduce AF vulnerability^23^. Moreover, sacubitril/valsartan was shown to reduce the expression of phosphorylated calmodulin-dependent protein kinase II (CaMKII), which is an established pro-arrhythmic molecule^24^.

Cardiac conduction velocity is closely related to voltage-dependent sodium currents, cardiomyocyte–cardiomyocyte gap junctional coupling, and muscle bundle anatomic structure^18,25^. Lowered sodium currents, reduced cardiomyocyte electric coupling, and atrial muscle bundle disorganization caused by fibrosis all reduce cardiac conduction and facilitate reentry. In addition, lowered I_CaL_, increased inward-rectifier K^+^ currents, and slow delayed rectifier K^+^ currents shorten APD and promote reentry^26–28^. A recent study demonstrated that sacubitril/valsartan increased I_CaL_ density in a rapid atrial pacing-induced rabbit AF model, which may contribute to inhibit the formation of reentry^29^.

Structural remodeling in AF progression is characterized primarily by increased atrial fibrosis and atrial enlargement. The major profibrotic molecules include angiotensin II and transforming growth factor β1 (TGFβ1), and the signaling pathways mainly involve Jun N-terminal kinase (JNK), mitogen-activated protein kinase (MAPK), and extracellular signal-related kinases^30,31^. In a rodent study, sacubitril/valsartan was found to suppress TGFβ1-Smad2/3, p-p38, and p-JNK signaling pathways, and reverse atrial fibrosis, thereby inhibiting AF progression^32^. Similarly, in a clinical setting, improved left atrial size was observed in HF AF patients^17,33^.

Over the past few decades, despite tremendous advances in our understanding of the electropathology of AF, the mechanisms underlying AF progression remain elusive. In addition to the general concern of fibrosis, fat accumulation, amyloidosis, and other still unidentified factors may be important for AF progression^34,35^. Besides, animal models induced by a specific single stimulus in the short term may limit the observation of complex mechanisms. Interventions that are successful in animal models often fail in clinical practice. In fact, clinical AF is usually the result of long-term complex pathophysiology. Therefore, the inhibition of AF progression by sacubitril/valsartan observed in the real-world has important clinical significance.

This study has several limitations. Firstly, this is a retrospective study, which inevitably includes bias in patient selection; our analysis should be considered hypothesis generating, and therefore further prospective studies are required. Secondly, subgroup analysis could not be conducted, due to limited subjects. Thirdly, the proportions of patients who achieved the target dose were 27.3% and 22.7% in sacubitril/valsartan and ARB groups, respectively. Although these numbers are not ideal, they reflect a real world clinical setting. Lastly, we used 7-days Holter monitoring to record the transition from paroxysmal to persistent AF. However, long-term (> 7 days) continuous electrocardiograph (ECG) monitoring can obtain more accurate information.

In summary, sacubitril/valsartan may be superior to ARB in reducing the risk of AF progression from paroxysmal to persistent in patients with paroxysmal AF who did not receive catheter ablation.

## Methods

### Study design and participants

This retrospective cohort study was conducted on consecutive patients with paroxysmal AF admitted at the Second Affiliated Hospital of Nanchang University between January 2017 and January 2022. Patients with paroxysmal AF were reviewed from the hospital’s electronic database. Exclusion criteria were: (1) patient who had received ablation therapy; (2) did not receive ARB or ARNI; (3) did not receive 7-day Holter monitoring; (4) previous history of cardiomyopathy or valvular; (5) chronic inflammatory disease; (6) connective tissue disease; (7) hyperthyroidism and (8) malignancy.

### Drug therapy

The first-line antiarrhythmic drug was propafenone (600 mg per day). However, amiodarone (200 mg per day) or sotalol (160 mg per day) was administered when propafenone was contraindicated. Rate control drugs, including beta receptor blockers, calcium channel blockers, and digoxin, were administered as necessary. The use of ARB or ARNI was based on the recommended guideline and physician’s choice, and the dosages were adjusted according to blood pressure, and individual tolerance^36,37^. The estimated risk of thromboembolism was calculated for each patient based on the CHA2DS2-VASc. Oral anticoagulants, including warfarin, dabigatran, and rivaroxaban, were recommended to prevent ischemic stroke in patients with a CHA2DS2-VASc score greater than 1 in males or greater than 2 in females.

### Data collection

General information on age, sex, BMI, duration of AF, and comorbidities was collected. Moreover, the blood pressure values of all patients were recorded at the time of the first outpatient visit or admission. Laboratory values, such as TC, triglyceride (TG), high density lipoprotein cholesterol (HDL-c), LDL-c, estimated glomerular filtration rate (eGFR), and BNP were also collected. Echocardiographic cardiac parameters such as LAD, and LVEF were measured. Other clinical data included the New York Heart Association functional classification, medications, and long-term ECG record.

### Patients follow-up and clinical outcomes

During the first year, outpatient follow-up was conducted every 1–3 months and every 6 months thereafter. Patients were advised to seek immediate clinic follow-up if AF-related symptoms occurred^36,37^. Twenty-four-hour Holter monitoring was conducted at each visit. Seven-day Holter monitoring was performed annually or whenever necessary. The risk of progression from paroxysmal to persistent AF was compared between paroxysmal patients treated with ARNI and those who received an ARB. Paroxysmal AF was defined as AF that spontaneously terminated or with intervention within 7 days, and persistent AF was defined as AF that lasted ≥ 7 days. Patients were censored if they discontinued ARNI or ARB therapy during the period of follow-up.

### Statement of ethics

This study was approved by the Medical Ethics Committee of the Second Affiliated Hospital of Nanchang University and met the standards of the Declaration of Helsinki. Informed consent was waived by the Medical Ethics Committee of the Second Affiliated Hospital of Nanchang University because of the retrospective nature of this study.

### Statistical analysis

Variables were expressed as means (standard deviations, [SD]) or median (IQR), and frequencies (proportions [%]). Between-group comparisons were conducted using Student's t- or the rank-sum test, and χ^2^- or the Fisher’s exact test. PSM was conducted using the nearest-neighbor method with a caliper width of 0.2 to reduce potential bias between ARNI and ARB. A standardized differences < 0.2 was considered to indicate acceptable balanced groups on a given covariate^38^. Survival analysis was performed in the matched cohorts to compare the risk of AF progression between groups. The HR, and 95% CI were computed. The proportional hazards assumption was checked using the Schoenfeld residuals. All data analyses were performed using RStudio version 1.1.414 (Boston, MA, USA) and Empower (http://www.empowerstats.com; X&Y Solutions, Inc., Boston, MA).

## Supplementary Information

Supplementary Tables.

## Data Availability

All data generated or analyzed during this study are included in this article. Further enquiries can be directed to the corresponding author.

## References

[1] Hindricks G, Potpara T, Dagres N, Arbelo E, Bax JJ, Blomstrom-Lundqvist C (2021). 2020 ESC guidelines for the diagnosis and management of atrial fibrillation developed in collaboration with the European Association for Cardio-Thoracic Surgery (EACTS): The task force for the diagnosis and management of atrial fibrillation of the European Society of Cardiology (ESC) developed with the special contribution of the European Heart Rhythm Association (EHRA) of the esc. Eur. Heart. J..

[2] Deng H, Bai Y, Shantsila A, Fauchier L, Potpara TS, Lip G (2017). Clinical scores for outcomes of rhythm control or arrhythmia progression in patients with atrial fibrillation: A systematic review. Clin. Res. Cardiol..

[3] Piccini JP, Passman R, Turakhia M, Connolly AT, Nabutovsky Y, Varma N (2019). Atrial fibrillation burden, progression, and the risk of death: A case-crossover analysis in patients with cardiac implantable electronic devices. Europace.

[4] Pascual-Figal D, Bayes-Genis A, Beltran-Troncoso P, Caravaca-Perez P, Conde-Martel A, Crespo-Leiro MG (2021). Sacubitril-valsartan, clinical benefits and related mechanisms of action in heart failure with reduced ejection fraction: A review. Front. Cardiovasc. Med..

[5] Gu J, Noe A, Chandra P, Al-Fayoumi S, Ligueros-Saylan M, Sarangapani R (2010). Pharmacokinetics and pharmacodynamics of LCZ696, a novel dual-acting angiotensin receptor-neprilysin inhibitor (ARNI). J. Clin. Pharmacol..

[6] Mcmurray JJ, Packer M, Desai AS, Gong J, Lefkowitz MP, Rizkala AR (2014). Angiotensin-neprilysin inhibition versus enalapril in heart failure. N. Engl. J. Med..

[7] Huo Y, Li W, Webb R, Zhao L, Wang Q, Guo W (2019). Efficacy and safety of sacubitril/valsartan compared with olmesartan in Asian patients with essential hypertension: A randomized, double-blind, 8-week study. J. Clin. Hypertens.

[8] Cheung DG, Aizenberg D, Gorbunov V, Hafeez K, Chen CW, Zhang J (2018). Efficacy and safety of sacubitril/valsartan in patients with essential hypertension uncontrolled by olmesartan: A randomized, double-blind, 8-week study. J. Clin. Hypertens..

[9] Dong Y, Xu Y, Ding C, Yu Z, Yu Z, Xia X (2022). Comparing the efficacy of angiotensin receptor-neprilysin inhibitor and enalapril in acute anterior STEMI patients after primary percutaneous coronary intervention: A prospective randomized trial. Cardiovasc. Diagn. Ther..

[10] Rezq A, Saad M, El NM (2021). Comparison of the efficacy and safety of sacubitril/valsartan versus ramipril in patients with ST-segment elevation myocardial infarction. Am. J. Cardiol..

[11] She J, Lou B, Liu H, Zhou B, Jiang GT, Luo Y (2021). ARNI versus ACEI/ARB in reducing cardiovascular outcomes after myocardial infarction. ESC. Heart. Fail..

[12] Ogawa H, Akao M (2022). Is progression from paroxysmal to sustained atrial fibrillation bad news?. Circ. J..

[13] de Vos CB, Pisters R, Nieuwlaat R, Prins MH, Tieleman RG, Coelen RJ (2010). Progression from paroxysmal to persistent atrial fibrillation clinical correlates and prognosis. J. Am. Coll. Cardiol..

[14] Kerr CR, Humphries KH, Talajic M, Klein GJ, Connolly SJ, Green M (2005). Progression to chronic atrial fibrillation after the initial diagnosis of paroxysmal atrial fibrillation: results from the Canadian registry of atrial fibrillation. Am. Heart. J..

[15] Pfeffer MA, Claggett B, Lewis EF, Granger CB, Kober L, Maggioni AP (2021). Angiotensin receptor-neprilysin inhibition in acute myocardial infarction. N. Engl. J. Med..

[16] Wang Q, Zhuo C, Xia Q, Jiang J, Wu B, Zhou D (2022). Sacubitril/valsartan can reduce atrial fibrillation recurrence after catheter ablation in patients with persistent atrial fibrillation. Cardiovasc. Drugs. Ther..

[17] Yang L, Zhang M, Hao Z, Wang N, Zhang M (2022). Sacubitril/valsartan attenuates atrial structural remodelling in atrial fibrillation patients. ESC. Heart. Fail..

[18] Heijman J, Voigt N, Nattel S, Dobrev D (2014). Cellular and molecular electrophysiology of atrial fibrillation initiation, maintenance, and progression. Circ. Res..

[19] Nattel S, Heijman J, Zhou L, Dobrev D (2020). Molecular basis of atrial fibrillation pathophysiology and therapy: A translational perspective. Circ. Res..

[20] Zellerhoff S, Pistulli R, Monnig G, Hinterseer M, Beckmann BM, Kobe J (2009). Atrial arrhythmias in long-QT syndrome under daily life conditions: A nested case control study. J. Cardiovasc. Electrophysiol..

[21] Lemoine MD, Duverger JE, Naud P, Chartier D, Qi XY, Comtois P (2011). Arrhythmogenic left atrial cellular electrophysiology in a murine genetic long QT syndrome model. Cardiovasc. Res..

[22] Bers DM (2014). Cardiac sarcoplasmic reticulum calcium leak: Basis and roles in cardiac dysfunction. Annu. Rev. Physiol..

[23] Cheng WH, Lugtu IC, Chang SL, Liu SH, Chen SA, Lo LW (2021). Effects of angiotensin receptor-neprilysin inhibitor in arrhythmogenicity following left atrial appendage closure in an animal model. Cardiovasc. Drugs. Ther..

[24] Chang PC, Wo HT, Lee HL, Lin SF, Chu Y, Wen MS (2020). Sacubitril/valsartan therapy ameliorates ventricular tachyarrhythmia inducibility in a rabbit myocardial infarction model. J. Card. Fail..

[25] Dobrev D, Carlsson L, Nattel S (2012). Novel molecular targets for atrial fibrillation therapy. Nat. Rev. Drug. Discov..

[26] Christ T, Boknik P, Wohrl S, Wettwer E, Graf EM, Bosch RF (2004). L-type Ca^2+^ current downregulation in chronic human atrial fibrillation is associated with increased activity of protein phosphatases. Circulation.

[27] Voigt N, Trausch A, Knaut M, Matschke K, Varro A, Van Wagoner DR (2010). Left-to-right atrial inward rectifier potassium current gradients in patients with paroxysmal versus chronic atrial fibrillation. Circ. Arrhythm. Electrophysiol..

[28] Caballero R, de la Fuente MG, Gomez R, Barana A, Amoros I, Dolz-Gaiton P (2010). In humans, chronic atrial fibrillation decreases the transient outward current and ultrarapid component of the delayed rectifier current differentially on each atria and increases the slow component of the delayed rectifier current in both. J. Am. Coll. Cardiol..

[29] Li LY, Lou Q, Liu GZ, Lv JC, Yun FX, Li TK (2020). Sacubitril/valsartan attenuates atrial electrical and structural remodelling in a rabbit model of atrial fibrillation. Eur. J. Pharmacol..

[30] Cardin S, Li D, Thorin-Trescases N, Leung TK, Thorin E, Nattel S (2003). Evolution of the atrial fibrillation substrate in experimental congestive heart failure: Angiotensin-dependent and -independent pathways. Cardiovasc. Res..

[31] Tan AY, Zimetbaum P (2011). Atrial fibrillation and atrial fibrosis. J. Cardiovasc. Pharmacol..

[32] Li SN, Zhang JR, Zhou L, Xi H, Li CY, Zhao L (2022). Sacubitril/valsartan decreases atrial fibrillation susceptibility by inhibiting angiotensin ii-induced atrial fibrosis through p-smad2/3, p-jnk, and p-p38 signaling pathways. J. Cardiovasc. Transl. Res..

[33] Desai AS, Solomon SD, Shah AM, Claggett BL, Fang JC, Izzo J (2019). Effect of sacubitril-valsartan vs enalapril on aortic stiffness in patients with heart failure and reduced ejection fraction: A randomized clinical trial. JAMA.

[34] Venteclef N, Guglielmi V, Balse E, Gaborit B, Cotillard A, Atassi F (2015). Human epicardial adipose tissue induces fibrosis of the atrial myocardium through the secretion of adipo-fibrokines. Eur. Heart. J..

[35] Rocken C, Peters B, Juenemann G, Saeger W, Klein HU, Huth C (2002). Atrial amyloidosis: An arrhythmogenic substrate for persistent atrial fibrillation. Circulation.

[36] Kario K, Shin J, Chen CH, Buranakitjaroen P, Chia YC, Divinagracia R (2019). Expert panel consensus recommendations for ambulatory blood pressure monitoring in Asia: The HOPE Asia network. J. Clin. Hypertens..

[37] Yancy CW, Jessup M, Bozkurt B, Butler J, Casey DJ, Colvin MM (2017). 2017 ACC/AHA/HFSA focused update of the 2013 ACCF/AHA guideline for the management of heart failure: A report of the American College of Cardiology/American Heart Association task force on clinical practice guidelines and the heart failure society of America. J. Card. Fail..

[38] Austin PC (2009). Balance diagnostics for comparing the distribution of baseline covariates between treatment groups in propensity-score matched samples. Stat. Med..

