# Supplementary material for: Angiotensin receptor-neprilysin inhibitor delays progression from paroxysmal to persistent atrial fibrillation
Source: Sci Rep. 2023 Feb 23;13:3140. doi: 10.1038/s41598-023-30349-w (PMC9950488; doi:10.1038/s41598-023-30349-w)
Supplement: Supplementary file 1 — Supplementary Tables. [file 41598_2023_30349_MOESM1_ESM.docx]

**Angiotensin receptor-neprilysin inhibitor delays progression from** **paroxysmal to** **persistent atrial fibrillation**

Youzheng Dong^1^^#^, Zhenyu Zhai^1#^, Jihong Wang^1#^, Zhen Xia^1^, Zirong Xia^1^, Bo Zhu^1^, Quanbing Dong^1^, Qing Li^1^, Juxiang Li^1
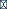
^

^1^ Department of Cardiovascular Medicine, the Second Affiliated Hospital of Nanchang University, No.1 of Minde Road, Nanchang, 330006, China

^#^ These authors contributed equally: Youzheng Dong, Zhenyu Zhai and Jihong Wang.

^
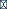
^**Corresponding author:** Juxiang Li, E-mail: [juxiang__li@163.com](mailto:juxiang__li@163.com); Department of Cardiovascular Medicine, the Second Affiliated Hospital of Nanchang University, No.1 of Minde Road, Nanchang, 330006, China.

**Table S1. The number of patients and** **time interval for each 24-hour Holter monitoring.**

| 24-hour Holter monitoring | ARB | ARNI | P-value |
| --- | --- | --- | --- |
| Holter 1 | (47) 30.00 (29.00-31.50) | (47) 30.00 (28.00-31.50) | 0.942 |
| Holter 2 | (47) 87.00 (60.50-93.00) | (47) 90.00 (64.00-114.50) | 0.264 |
| Holter 3 | (47) 153.00 (126.00-175.50) | (47) 157.00 (145.00-179.50) | 0.316 |
| Holter 4 | (45) 216.00 (201.00-242.00) | (47) 236.00 (205.50-272.50) | 0.170 |
| Holter 5 | (38) 457.00 (395.00-496.50) | (45) 477.00 (453.00-515.00) | 0.141 |
| Holter 6 | (33) 661.00 (563.00-813.00) | (31) 680.00 (593.50-836.00) | 0.304 |
| Holter 7 | (13) 967.00 (925.00-994.00) | (15) 976.00 (805.00-1043.00) | 0.872 |
| Holter 8 | (5) 1240.00 (1086.00-1400.00) | (2) 1054.00 (1038.50-1069.50) | 0.245 |
| Abbreviations: ARB=angiotensin receptor blocker; ARNI=Angiotensin receptor neprilysin inhibitor. | | | |

**Table S2. The number of patients and time interval for each** **7-day Holter monitoring.**

| 7-day Holter monitoring | ARB | ARNI | P-value |
| --- | --- | --- | --- |
| Holter 1 | (47) 304.00 (272.00-357.00) | (47) 309.00 (293.00-352.50) | 0.586 |
| Holter 2 | (28) 650.00 (605.75-747.50) | (24) 663.50 (628.50-738.75) | 0.388 |
| Holter 3 | (8) 987.50 (975.00-1022.50) | (7) 998.00 (922.00-1020.50) | 0.862 |
| Holter 4 | (3) 1273.00 (1238.50-1313.00) | (1) 1462.00 (1462.00-1462.00) | 0.180 |
| Abbreviations: ARB=angiotensin receptor blocker; ARNI=Angiotensin receptor neprilysin inhibitor. | | | |

**Table S3. Changes of LVEF and LAD before and after treatment.**

|  | ARB | | | ARNI | | |  |
| --- | --- | --- | --- | --- | --- | --- | --- |
| Variables | Baseline | After treatment | Δ from baseline | Baseline | After treatment | Δ from baseline | Between-group difference in change from baseline (P-value) |
| Before PSM |  |  |  |  |  |  |  |
| LAD (mm) | (113) 36.63 ± 5.39 | (95) 36.97 ± 4.51 | (95) 0.29 ± 3.41 | (57) 38.91 ± 6.57 | (50) 37.38 ± 5.61 | (50) -1.48 ± 3.12 | 0.006 |
| LVEF (%) | (113) 62.52 ± 8.57 | (95) 63.96 ± 8.58 | (95) 1.68 ± 3.51 | (57) 57.79 ± 11.42 | (50) 62.22 ± 10.07 | (50) 4.86 ± 3.30 | <0.001 |
| After PSM |  |  |  |  |  |  |  |
| LAD (mm) | (47) 37.23 ± 5.61 | (39) 37.46 ± 4.38 | (39) 0.38 ± 3.75 | (47) 37.79 ± 5.79 | (40) 36.80 ± 5.32 | (40) -1.00 ± 2.87 | 0.109 |
| LVEF (%) | (47) 61.21 ± 10.82 | (39) 61.49 ± 10.17 | (39) 0.72 ± 3.39 | (47) 59.91 ± 10.73 | (40) 63.55 ± 9.89 | (40) 4.50 ± 3.00 | <0.001 |
| Abbreviations: LVEF=left ventricle ejection fraction; LAD=left atrial diameter; ARB=angiotensin receptor blocker; ARNI=Angiotensin receptor neprilysin inhibitor; PSM= propensity-score matching. | | | | | | | |
